# Prevalence and clinical associations of cerebral atrophy in systemic lupus erythematosus

**DOI:** 10.1136/lupus-2026-002005

**Published:** 2026-07-20

**Authors:** Woon Shen Teo, Choung Min Ng, Lydia Say Lee Pok, Farhana Fadzli, Kartini Rahmat, Jasmin Raja

**Affiliations:** 1 Rheumatology, Universiti Malaya Faculty of Medicine, Federal Territory of Kuala Lumpur, Malaysia; 2 Institute of Mathematical Science, Universiti Malaya, Federal Territory of Kuala Lumpur, Malaysia; 3 Biomedical Imaging, Universiti Malaya, Federal Territory of Kuala Lumpur, Malaysia

**Keywords:** Lupus Erythematosus, Systemic, Autoimmune Diseases, Prevalence

## Abstract

**Objectives:**

To determine the prevalence of cerebral atrophy in a multi-ethnic systemic lupus erythematosus (SLE) cohort and to identify its associated clinical factors.

**Methods:**

In this cross-sectional study (2024–2025), adults fulfilling the 2019 European League Against Rheumatism/American College of Rheumatology (EULAR/ACR) classification criteria for SLE at University Malaya Medical Centre were recruited. Demographic, clinical, serological, vascular and treatment data were collected. Disease activity and cumulative damage were assessed using the SLE Disease Activity Index 2000 (SLEDAI-2K) and the Systemic Lupus International Collaborating Clinics/American College of Rheumatology Damage Index (SDI). Non-contrast CT brain scans were performed with cerebral atrophy graded using the validated Global Cortical Atrophy (GCA) score. Cognitive function was evaluated using the Montreal Cognitive Assessment (MoCA). Fisher’s exact test and χ^2^ test were used to test the association between two categorical variables where appropriate, while group differences were tested using the Mann-Whitney U test. Univariable and multivariable logistic regression analyses were considered to identify variables that were significantly associated with cerebral atrophy.

**Results:**

Seventy patients (92.9% female; median age 40 (IQR 31.75–50.25) years; median disease duration 12 (IQR 4–19) years were included. Cerebral atrophy was present in 52.9%, predominantly mild (75.7%). From univariate comparisons, patients with cerebral atrophy were significantly older in age, had longer disease duration, lower education, higher SDI and poorer MoCA scores. Multivariable analysis showed that older age (OR 1.10 per year, 95% CI 1.04 to 1.18) and neuropsychiatric SLE (NPSLE) (OR 4.58, 95% CI 1.05 to 24.07) contributed to increased odds of cerebral atrophy, while higher educational attainment was linked with reduced odds of cerebral atrophy (OR 0.22, 95% CI 0.06 to 1.03).

**Conclusions:**

Cerebral atrophy is common in SLE and independently associated with age, education, NPSLE and cognitive impairment, reflecting neuroinflammatory and neurodegenerative mechanisms. These findings underscore the importance of cognitive screening and targeted monitoring of high-risk patients.

**WHAT IS ALREADY KNOWN ON THIS TOPIC**

- Cerebral atrophy is one of the most frequently reported neuroimaging abnormalities in patients with SLE.
- Previous studies have suggested associations with disease duration, cumulative corticosteroid exposure, disease activity and neuropsychiatric SLE (NPSLE); however, findings have been inconsistent.
- Most available data are derived from heterogenous populations or studies focused predominantly on overt NPSLE, with many studies lacking clear attribution criteria for NPSLE, thereby limiting generalisability.

**WHAT THIS STUDY ADDS**

- This study provides data on the prevalence of cerebral atrophy in a well-characterised SLE cohort, including patients without overt neuropsychiatric manifestations as well as patients with NPSLE defined using the stricter Systemic Lupus International Collaborating Clinics Damage Index Attribution Model (A-criteria).
- We identified specific clinical and disease-related factors associated with cerebral atrophy, offering insight into potential risk markers.
- This study adds evidence from a multi-ethnic Asian SLE population, addressing an important gap in existing literature.
- Our findings demonstrated an association between cerebral atrophy and lower Montreal Cognitive Assessment scores, suggesting that structural brain changes in SLE may have clinically relevant cognitive consequences.

**HOW THIS STUDY MIGHT AFFECT RESEARCH, PRACTICE OR POLICY**

- The high prevalence of cerebral atrophy identified in our cohort underscores the need for greater awareness of subclinical cerebral involvement in SLE, even in patients without neuropsychiatric symptoms.
- Identification of associated clinical factors emphasises the need for aggressive disease control and vascular risk factors to potentially mitigate long-term structural brain changes in SLE.
- These findings provide a rationale for longitudinal studies to better define the progression and clinical consequences of cerebral atrophy in SLE.

## Introduction

 Neuropsychiatric systemic lupus erythematosus (NPSLE) is a challenging manifestation of lupus, encompassing a spectrum of neuroimaging abnormalities, including white matter hyperintensities, lacunar infarcts, inflammatory-like lesions and cerebral atrophy.^1 2^ Many findings are non-specific, overlapping with age-related, vascular or other autoimmune pathologies. Among these, cerebral atrophy is frequently reported, with prevalence estimates ranging from 32% to 71%.^3^,^7^ It is strongly associated with cognitive dysfunction, a recognised case definition under the 1999 American College of Rheumatology (ACR) Nomenclature and Case Definitions for NPSLE.^8^,^13^ Conversely, cerebral atrophy in systemic lupus erythematosus (SLE) may also occur in the absence of overt neuropsychiatric symptoms or cognitive dysfunction, making the clinical significance in this context unclear.^14^ Although not fully understood, cerebral atrophy may plausibly be a precursor to future neurocognitive impairment.

Two key gaps exist in the literature. First, there is a paucity of data on the prevalence of cerebral atrophy in patients with SLE in Asia, particularly in Southeast Asia. The second major research gap concerns the inconsistency of findings regarding the clinical and immunological associations of cerebral atrophy. While some studies have identified associations between cerebral atrophy and certain disease-related variables such as disease activity, duration of disease, presence of antiphospholipid and anti-Ro antibodies, and glucocorticoid exposure, others have failed to demonstrate such links.^1^,^1315^

This study aimed to address these research gaps. The first objective was to determine the prevalence of cerebral atrophy among patients with SLE in Malaysia using a multi-ethnic general SLE cohort, beyond those with neuropsychiatric manifestations. Our second aim was to determine the clinical and laboratory associations of cerebral atrophy using standardised assessment tools, including the SLE Disease Activity Index 2000 (SLEDAI-2K),^18^ Systemic Lupus International Collaborating Clinics (SLICC)/ACR Damage Index (SDI),^19^ Montreal Cognitive Assessment (MoCA)^20^ and Global Cortical Atrophy (GCA) scores.^21^

Through a better understanding of these associations, this study aimed to explore and identify SLE subgroups at a higher risk of developing cerebral atrophy for targeted interventions.

## Methods

An exploratory cross-sectional study was conducted from July 2024 to July 2025 at University Malaya Medical Centre, a tertiary university hospital in Malaysia. Adults who fulfilled the 2019 European League Against Rheumatism (EULAR)/ACR classification criteria for SLE were consecutively recruited from our lupus outpatient clinic between 2024 and 2025. Both new and follow-up outpatients were considered for inclusion. The exclusion criteria were age above 60 years, pregnancy and refusal to participate. Patients above 60 years were excluded to minimise confounding from age-related cerebral atrophy, which could obscure the association between SLE-related factors and brain changes; however, we acknowledge that this may limit generalisability to older populations. The estimated sample size was determined to be 49 patients, based on the observed prevalence of up to 71% from the literature. This sample size was calculated using an estimated moderate effect size of 0.4 to achieve 80% power, at a 5% significance level, in rejecting the null hypothesis of a 51.5% prevalence of patients with SLE in Malaysia, which represents the midpoint of the prevalence estimates ranging from 32% to 71% reported in the literature.^3^,^7^ Data collection included demographics, disease duration (interval from SLE onset to computed tomography (CT) brain study), organ involvement, serological and treatment data, comorbidities, stroke history and vascular risk factors, including the presence of diabetes mellitus, dyslipidaemia, obesity, hypertension, ischaemic heart disease and smoking status. People who previously smoked and people who smoke were classified into a single ‘smoker’ category, while people who have never smoked were defined as individuals who had smoked fewer than 100 cigarettes in their lifetime.^22^

Vascular risk factors were defined using standard clinical and biochemical criteria as follows: diabetes mellitus (defined as random blood glucose level of >11.1 mmol/L, fasting blood glucose >7.0 mmol/L, glycated haemoglobin >6.5% or current use of antidiabetic drugs), past or current smoking, dyslipidaemia (low-density lipoprotein cholesterol >3.64 mmol/L, high-density lipoprotein cholesterol <0.91 mmol/L, triglyceride >1.7 mmol/L or usage of lipid-lowering therapy), obesity (body mass index >25 kg/m^2^), history of ischaemic heart disease and hypertension.^23^

Organ involvement, encompassing both past and present manifestations, was defined using predefined clinical and laboratory criteria consistent with 2019 EULAR/ACR SLE classification criteria.^24^ Mucocutaneous involvement included non-scarring alopecia, oral ulcers, acute cutaneous lupus, subacute cutaneous lupus or discoid rash. Musculoskeletal involvement was defined as arthritis affecting two or more peripheral joints characterised by swelling or effusion. Serositis included pleuritis, pericarditis or radiological evidence of effusion. Renal involvement was defined as biopsy-proven lupus nephritis, or proteinuria >0.5 g/24 hours. Haematological involvement included haemolytic anaemia, leucopenia (<4000/μL) or thrombocytopenia (<100,000/μL). Positive antiphospholipid antibody is defined by the presence of anticardiolipin antibodies at medium or high titre (>40 Units) or positive lupus anticoagulant. Antiphospholipid syndrome (APS) was defined according to the 2023 ACR/EULAR criteria, requiring at least one positive antiphospholipid antibody within three years of a related clinical event, supported by weighted criteria across six clinical and two laboratory domains.^25^

As for the case definitions for NPSLE, NPSLE was initially screened using the 1999 ACR case definitions and subsequently evaluated with the more stringent SLICC and Italian study group attribution models for NPSLE.^26 27^ The final attribution of NPSLE was determined after careful exclusion of alternative aetiologies by reviewing multidisciplinary input, including neurology, guided by SLICC decision rules and the NPSLE attribution model.

Disease activity and damage were assessed using the SLEDAI-2K and SDI Damage Index, respectively,^18 19^ and cognitive function was evaluated using the MoCA.^20^ Cumulative steroid dose was determined through detailed review of electronic medical records and calculated from the time of SLE diagnosis to the date of the CT brain study, with all doses converted into prednisolone-equivalent dose.

All participants underwent a standard non-contrast CT brain scan with a 3 mm slice thickness at 120 kV. The choice of CT imaging over MRI was due to the wider availability, shorter acquisition time and lower cost in our centre, which allowed inclusion of all consecutive SLE outpatients during the study period. Cerebral atrophy was assessed using the validated GCA scale, a semi-quantitative visual rating system based on assessment of 13 brain regions, with sulcal widening and gyral volume loss graded from 0 (normal) to 3 (severe).^21^ Cerebral atrophy was defined as a GCA score ≥1, with scores of 1, 2 and 3 representing mild, moderate and severe atrophy, respectively. The GCA scale has been validated for use on both CT and MRI, with studies demonstrating good inter-rater reliability and comparable performance for detecting global atrophy on CT scans.^28^ All scans were independently reviewed by a single neuroradiologist blinded to the clinical outcomes to minimise interobserver variability.

### Patient and public involvement

Patients and members of the public were not involved in the design, conduct, reporting or dissemination plans of this research.

### Statistical analysis

Continuous variables were summarised as median (IQR) and categorical variables as frequencies (%). Group differences were assessed using the Mann-Whitney U test for continuous data while associations between two categorical variables were tested using χ^2^ or Fisher’s exact. Univariable logistic regression was used to identify factors associated with cerebral atrophy. Variables with p values <0.30 from the univariable logistic regression were considered in the subsequent bidirectional stepwise multivariable logistic regression analysis, where these variables were iteratively added or removed to achieve the lowest Akaike information criterion value for the final model selection. The final multivariable logistic model was assessed for overall significance using the likelihood ratio test, as well as for goodness-of-fit and predictive accuracy using the Hosmer-Lemeshow test and Brier score. Statistical significance was set at p<0.05. Analyses were performed using the SPSS (V.29.0; IBM) and R statistical software (V.4.4.2).

## Results

**Figure 1:**
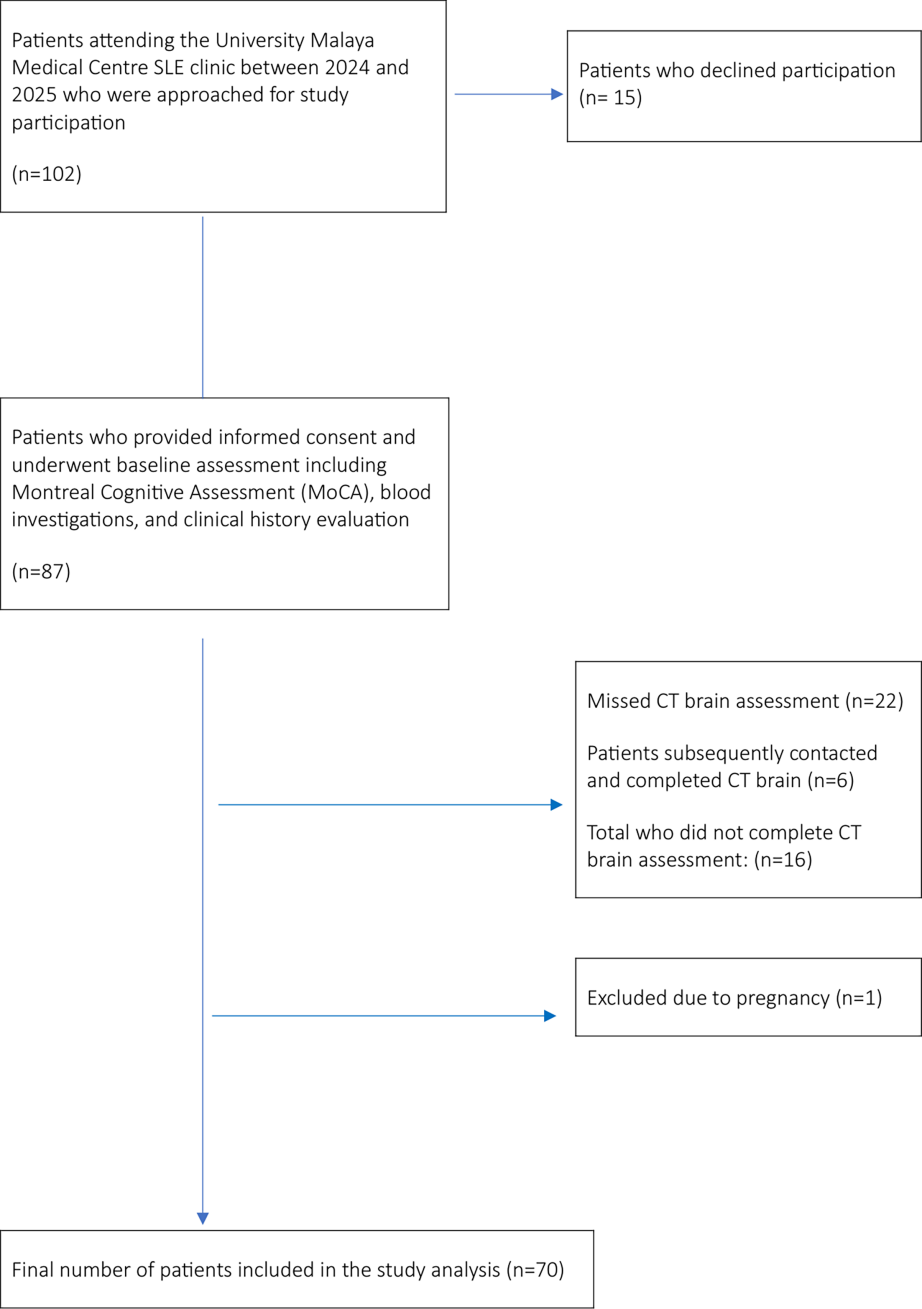
Screening process of patients with SLE in SLE clinic, University Malaya Medical Centre from 2024 to 2025.

A total of 102 patients were approached for participation in the study. Of these, 15 declined consent, citing scheduling conflicts related to work commitments and concerns regarding potential radiation exposure. In total, 87 patients were recruited into the study and underwent basic blood tests and MoCA assessment. Out of these consenting patients, 16 defaulted their CT imaging appointments, while one newly pregnant patient was excluded (figure 1). The remaining 70 patients were included, the majority being female (n=65; 92.9%) with a median age of 40 years (IQR 31.75–50.25), and a median disease duration of 12 years (IQR 4–19) (table 1). Most patients were of Chinese ethnicity (55.7%) followed by Malay (32.9%). Half had attained high education levels. Vascular comorbidities were present in 51.4% and 10.0% had a history of stroke. Median disease duration of this cohort was 12 years (4–19 years), with patients having a median disease activity SLEDAI-2K score of 4 (2–8) and SDI Damage Index score of 0 (0–1) (table 2).

**Table 1:** Demographics of the patients with SLE

Demographics	All (n=70)	No cerebral atrophy (n=33)	With cerebral atrophy (n=37)	P value*
Age, years	40.00 (31.75–50.25)	37.00 (30.50–43.00)	47.00 (36.50–54.50)	<0.001
Age at diagnosis, years	26.50 (21.00–35.50)	25.00 (22.50–31.50)	30.00 (20.50–38.00)	0.406
Gender				
Female	65 (92.9%)	33 (100%)	32 (86.5%)	0.056†
Male	5 (7.1%)	0 (0%)	5 (13.5%)	
Ethnicity				
Chinese	39 (55.7%)	16 (48.5%)	23 (62.2%)	0.603
Indian	5 (7.1%)	2 (6.1%)	3 (8.1%)	
Malay	23 (32.9%)	14 (42.4%)	9 (24.3%)	
Others	3 (4.3%)	1 (3.0%)	2 (5.4%)	
Education level				
High	35 (50.0%)	23 (69.7%)	12 (32.4%)	0.002
Medium/Low	35 (50.0%)	10 (30.3%)	25 (67.6%)	
Occupation				
Professional	18 (26.1%)	12 (36.4%)	6 (16.7%)	0.270
Support	35 (50.7%)	15 (45.5%)	20 (55.6%)	
Student	4 (5.8%)	2 (6.1%)	2 (5.6%)	
Unemployed	12 (17.4%)	4 (12.1%)	8 (22.2%)	

Patient demographics and clinical variables were reported as medians with IQRs between the first (25th) and third (75th) quartiles. Frequencies (%) were presented for categorical or ordinal variables. The mean age of the 70 patients was 40.53 years, with an SD of 11.06 years.

* Comparison between patients with and without cerebral atrophy. Mann-Whitney U test for continuous variables. Pearson’s χ2 test for categorical variables unless stated otherwise.

† Fisher’s exact test was performed.

**Table 2:** Clinical characteristics of the patients with SLE

Characteristics	All (n=70)	No cerebral atrophy (n=33)	With cerebral atrophy (n=37)	P value*
Disease duration, years	12.00 (4.00–19.00)	7.00 (3.00–17.50)	16.00 (7.25–22.25)	0.011
Renal involvement	37 (52.9%)	15 (45.5%)	22 (59.5%)	0.241
Haematological	37 (52.9%)	18 (54.5%)	19 (51.4%)	0.789
Arthritis	45 (64.3%)	23 (69.7%)	22 (59.5%)	0.372
Mucocutaneous	48 (68.6%)	24 (72.7%)	24 (64.9%)	0.479
Serositis	6 (8.6%)	3 (9.1%)	3 (8.1%)	>0.99†
NPSLE	18 (25.7%)	5 (15.2%)	13 (35.1%)	0.056
APS	12 (17.1%)	3 (9.1%)	9 (24.3%)	0.091
Vascular comorbidity	36 (51.4%)	13 (39.4%)	23 (62.2%)	0.057
Stroke	7 (10.0%)	1 (3.0%)	6 (16.2%)	0.110†
SLEDAI-2K	4 (2–8)	4 (2–9)	4 (0–8)	0.436
SDI	0 (0–1)	0 (0–1)	1 (0–2)	0.005
Low complements (C3/C4)	37 (52.9%)	17 (51.5%)	20 (54.1%)	0.832
Anti ds-DNA (IU/mL)	339.00 (71.50–603.50)	459.00 (138.00–671.00)	168.00 (40.00–412.50)	0.018
Lupus anticoagulant	11 (25.0%)	4 (16.0%)	7 (36.8%)	0.164†
Anticardiolipin antibody	12 (31.5%)	7 (31.8%)	5 (31.3%)	0.970
Anti-RNP	24 (49.0%)	14 (51.9%)	10 (45.5%)	0.656
Anti-Smith	19 (38.8%)	13 (48.1%)	6 (27.3%)	0.136
Anti-SSA	25 (51.0%)	14 (51.9%)	11 (50.0%)	0.897
Anti-SSB	6 (12.2%)	4 (14.8%)	2 (9.1%)	0.678†
Anti-Ro52	10 (23.8%)	3 (13.0%)	7 (36.8%)	0.143†
HCQ	63 (90.0%)	31 (93.9%)	32 (86.5%)	0.434†
DMARDs	31 (44.3%)	15 (45.5%)	16 (43.2%)	0.853
CYC	12 (17.1%)	5 (15.2%)	7 (18.9%)	0.676
RTX	10 (14.3%)	5 (15.2%)	5 (13.5%)	>0.99†
Cumulative prednisolone (g)	22.46 (4.41–61.11)	21.14 (4.33–67.53)	26.87 (4.45–63.40)	0.874
MoCA	26.00 (25.00–27.00)	27.00 (25.00–28.00)	26.00 (24.00–27.00)	0.004

Patient demographics and clinical variables were reported as median with IQRs between the first (25th) and third (75th) quartiles. Frequencies (%) were presented for categorical or ordinal variables.

* Comparison between patients with and without cerebral atrophy. Mann-Whitney U test for continuous variables. Pearson’s χ2 test for categorical variables.

† Fisher’s exact test was performed.

Anti-dsDNA, antidouble-stranded DNA; Anti-RNP, antiribonucleoprotein; Anti-SSA, anti-Sjögren's syndrome related antigen A; Anti-SSB, anti-Sjögren's syndrome related antigen B; APS, antiphospholipid syndrome; C3, complement 3; C4, complement 4; CYC, cyclophosphamide; DMARD, disease-modifying antirheumatic drug; HCQ, hydroxychloroquine; MoCA, Montreal Cognitive Assessment score; NPSLE, neuropsychiatric SLE; RTX, rituximab; SDI, Systemic Lupus International Collaborating Clinics Damage Index; SLEDAI-2K, SLE Disease Activity Index 2000.

With regard to the clinical characteristics, 25.7% of the patients had NPSLE while 17.1% had APS. Antiphospholipid antibody positivity was observed, with anticardiolipin antibodies present in 31.5% and lupus anticoagulant in 25.0% of patients. The median cumulative prednisolone use was 22.5 g while median MoCA score of this cohort was 26. With regard to medications, 90.0% of patients were on hydroxychloroquine, 44.3% were on conventional immunosuppressants, while 14.3% and 17.1% had a history of receiving rituximab and cyclophosphamide, respectively.

Cerebral atrophy was observed in 52.9% (n=37) of the patients. Among those with atrophy, 24.3% exhibited moderate-to-severe changes. Patients with cerebral atrophy were significantly older than those without (median age 47 vs 37 years; p<0.001) and had a longer disease duration (median 16 vs 7 years; p=0.011). Education level appeared significantly associated with the presence of cerebral atrophy, with 65.7% of highly educated patients showing no atrophy, whereas 72.7% of patients with low-medium level education had cerebral atrophy (p=0.002). A higher proportion of patients with vascular comorbidities and stroke exhibited cerebral atrophy, although these did not reach statistical significance (p=0.057 and 0.110, respectively) (table 2).

The distribution of GCA scores among patients with cerebral atrophy are as follows: out of the 37 patients with atrophy, 28 patients (75.7%) had grade 1 (mild), seven patients (18.9%) had grade 2 (moderate) and two patients (5.4%) had grade 3 (severe) cerebral atrophy. From table 2, patients with cerebral atrophy demonstrated higher cumulative organ damage (SDI scores), lower antidouble-stranded DNA (anti-dsDNA) antibody levels and poorer cognitive performance reflected by lower MoCA scores compared with those without atrophy (p=0.005, 0.018 and 0.004, respectively). There was no significant difference in disease activity, as measured by SLEDAI-2K scores, between patients with cerebral atrophy and without atrophy.

Similarly, no significant association was observed between cerebral atrophy and the presence of specific autoantibodies, including antiphospholipid markers such as lupus anticoagulant or anticardiolipin antibodies (p=0.164 and 0.970, respectively). Cumulative glucocorticoid dose, as well as the use of hydroxychloroquine, immunosuppressants and biologics, did not differ significantly between the groups. In addition, no significant associations were found between individual organ manifestations and cerebral atrophy. A higher proportion of patients with NPSLE and APS showed cerebral atrophy, suggesting a trend that did not reach statistical significance (p=0.056 and 0.091, respectively) (table 2).

From table 3, univariable logistic regression identified age (OR 1.09, 95% CI 1.03 to 1.14), education level (OR 0.21, 95% CI 0.08 to 0.57), MoCA total score (OR 0.77, 95% CI 0.62 to 0.95), disease duration (OR 1.05, 95% CI 0.99 to 1.12) and SDI (OR 2.16, 95% CI 1.19 to 3.91) as significantly associated with cerebral atrophy, with borderline significance for vascular comorbidity, NPSLE and anti-dsDNA. Subsequently, the multivariable stepwise logistic regression model revealed that age, education level, NPSLE and MoCA total score were independently associated with the presence of cerebral atrophy in patients with SLE after adjusting for other covariates.

**Table 3:** Logistic regression analyses for predictive factors in development of cerebral atrophy in patients with SLE

Factors	Development of cerebral atrophy			
	Univariable analysis		Multivariable analysis	
	OR (95% CI)	P value	OR (95% CI)	P value
Age, years	1.09 (1.03 to 1.14)	0.002	1.10 (1.04 to 1.18)	0.002
Education level (high)	0.21 (0.08 to 0.57)	0.002	0.22 (0.06 to 1.03)	0.014
NPSLE	3.03 (0.95 to 9.74)	0.062	4.58 (1.05 to 24.07)	0.053
MoCA total score	0.77 (0.62 to 0.95)	0.016	0.82 (0.63 to 1.03)	0.108
Disease duration, years	1.05 (0.99 to 1.12)	0.009		
SDI	2.16 (1.19 to 3.91)	0.011		
Vascular comorbidity	2.53 (0.96 to 6.63)	0.059		
Anti-dsDNA	0.999 (0.997 to 1.00)	0.065		
Stroke	6.19 (0.70 to 54.46)	0.100		
APS	3.21 (0.79 to 13.09)	0.103		
Lupus anticoagulant	3.06 (0.74 to 12.65)	0.122		
Age at diagnosis, years	1.08 (1.02 to 1.14)	0.212		
Renal involvement	1.76 (0.68 to 4.55)	0.243		
Cumulative prednisolone (g)	1.001 (0.999 to 1.002)	0.335		
SLEDAI-2K	0.99 (0.91 to 1.07)	0.795		

The multivariable logistic regression model was significant compared with the null model (likelihood ratio test: χ2=28.88, df=4, p<0.001), with adequate goodness-of-fit (Hosmer-Lemeshow test: p=0.488) and acceptable overall predictive accuracy (Brier score=0.164).

anti-dsDNA, antidouble-stranded DNA; APS, antiphospholipid syndrome; MoCA, Montreal Cognitive Assessment; NPSLE, neuropsychiatric SLE; SDI, Systemic Lupus International Collaborating Clinics Damage Index; SLEDAI-2K, SLE Disease Activity Index 2000.

Specifically, after adjusting for other covariates, each 1 year increase in age was associated with 10% higher odds of cerebral atrophy (OR 1.10, 95% CI 1.04 to 1.18); patients with high education had 78% lower odds compared with those with medium or low education (OR 0.22, 95% CI 0.06 to 1.03); NPSLE conferred 4.58-fold increased odds of developing cerebral atrophy than those without NPSLE (OR 4.58, 95% CI 1.05 to 24.07) and each one-point increase in MoCA score was associated with an 18% reduction in the odds of cerebral atrophy (OR 0.82, 95% CI 0.63 to 1.03).

## Discussion

The prevalence of cerebral atrophy in our SLE patient cohort (53%) aligns with the global estimates of patients with SLE ranging from 32% to 71%.^3^,^7^ Out of the 37 patients with cerebral atrophy, most patients (76%) exhibited mild atrophy, consistent with findings from previous cohorts.^5^ Notably, this study represents one of the few investigations in a multi-ethnic Southeast Asian SLE population. The broad variation in reported prevalence across studies is largely attributed to the inconsistent definitions and heterogeneous neuroimaging criteria used to define cerebral atrophy. In this study, we used the GCA scale, a rapid and validated screening tool that has been shown to correlate closely with MRI-derived measures of cortical atrophy.^21 28^ In contrast to many earlier studies that focused on patients with overt neuropsychiatric symptoms, our cohort included a broader SLE population, improving generalisability.

Age demonstrated the strongest independent association with cerebral atrophy, with each additional year increasing the odds by 10% (OR 1.10). While cerebral atrophy is a recognised feature of normal ageing^29^, our findings suggest that increasing age was significantly associated with brain structural changes within the SLE cohort. This observation is notable, given the relatively young median age of our cohort (40 years) and the exclusion of individuals aged >60 years. However, given the absence of a non-SLE control group, our findings should be interpreted as demonstrating an association between age and cerebral atrophy within the SLE cohort, rather than evidence of accelerated atrophy relative to the general population. Consistent with this observation, similar age-related associations have also been reported by Petri *et al*,^10^ with supporting evidence from other cohorts.^13^

Longer disease duration was also associated with atrophy, consistent with evidence from Brazilian cross-sectional and longitudinal studies, which support the concept of a cumulative accrual of damage driven by chronic autoantibody-mediated inflammation.^8 9 15^ However, the literature remains heterogeneous; some cohorts have not observed this association^5^ and studies in newly diagnosed SLE have reported the presence of atrophy even at disease onset,^7 10 11^ suggesting additional mechanisms beyond cumulative inflammation may contribute to early structural changes.

Building on these observations, our study further identified positive associations with additional variables, including higher SDI scores, reinforcing the role of accrued systemic damage. The relationship between higher SDI scores and the cerebral atrophy is supported by previous studies^11 13^ and aligns with the findings by Appenzeller *et al*^8 9^; their cross-sectional study did not show a significant association between SDI and atrophy, whereas their longitudinal study reported a positive association, reinforcing the concept of cumulative autoantibody-mediated damage. Future studies with larger cohorts could further clarify the relative contributions of neuropsychiatric-specific damage indices.^13 16^

Additionally, an inverse association was observed between ds-DNA titres and atrophy in our cohort, although this did not persist in multivariable model analysis. Given the fluctuating nature of dsDNA levels and the cross-sectional design, this may support the concept of a transition towards a chronic damage phenotype in longstanding disease, characterised by lower serological activity but accumulated neuroinflammatory injury and neurodegeneration.

No significant associations were found between antiphospholipid antibodies or APS and atrophy, consistent with previous studies.^4 10 17^ The small proportion of APS in our sample may have limited power to detect associations. This may reflect a true lack of association, although the small number of APS cases in our cohort limits firm conclusions, and any potential effect may be limited to higher-risk serological subgroups that were underrepresented in this cohort.

From our exploratory analyses, we found no significant associations between cerebral atrophy and autoantibodies, organ system manifestations, immunosuppressive therapies, vascular comorbidities or stroke. To minimise confounding, patients with major stroke were excluded. Similar findings from a Japanese cohort suggest that structural brain changes in SLE may primarily reflect small-vessel pathology rather than conventional macrovascular risk factors.^30^ Although vascular comorbidities and prior stroke were not independently associated with the presence of atrophy, the potential contribution of vascular factors to brain structural changes in SLE warrants further investigation.

The relationship between cumulative corticosteroid exposure and cerebral atrophy remains a highly contentious topic. In our study, we observed no association between cumulative prednisolone dose and atrophy, consistent with previous reports.^4 6 8^ Although one study reported greater atrophy in steroid-treated patients compared to healthy controls, comparisons between steroid-treated and steroid-naïve patients with SLE did not reveal significant differences, suggesting confounding by disease severity.^5^ Overall, interpretation is limited by substantial confounding, as higher steroid doses are typically prescribed for more active or severe disease, complicating the disentanglement of treatment effects from disease-related neuroinflammation.

NPSLE demonstrated an association with atrophy, with meaningful clinical relevance, although there was no statistical significance. A notable strength of our study is the application of the more stringent SLICC Attribution Model (A-criteria), thereby reducing potential misclassification inherent in the broader ACR definitions, commonly employed by many other studies.^8^,^13^ This methodological rigour enhances the specificity of our findings and suggests a potential association between NPSLE and structural brain changes. The biological plausibility of this relationship is supported by multiple converging mechanisms, including inflammatory, ischaemic and antibody-mediated pathways that can lead to neuronal loss and cortical atrophy.^37 8 12^,^14^

Cognitive function was independently associated with atrophy, with lower MoCA scores and lower educational attainment emerging as significant predictors. This aligns with previous studies linking cognitive dysfunction with structural brain changes.^4 8 12 13 17^ MoCA has increasingly been recognised as a practical and sensitive screening tool for cognitive impairment in SLE, offering advantages in brevity compared with the full ACR-SLE neuropsychological battery.^31^ The link between cognitive impairment and lower educational attainment further suggests that both biological vulnerability and cognitive reserve contribute to neurological outcomes. A limitation of our study is the absence of a healthy control group and the lack of assessment for psychiatric comorbidities, which may act as confounders influencing cognitive performance. Additionally, the relatively small sample size from a single-centre study and the absence of longitudinal follow-up may limit the generalisability of our findings and preclude assessment of temporal changes in cerebral atrophy as well as potential effects of ethnic variation. It should also be highlighted that there is a potential for overfitting in the multivariable logistic regression model due to the small sample size. Accordingly, the observed associations between age, clinical factors and cerebral atrophy in our SLE cohort should be interpreted in the context of these study limitations. External validation in larger cohorts is required.

In conclusion, cerebral atrophy was detected in more than half of our relatively young cohort, including patients without a diagnosis of NPSLE, suggesting that subclinical cerebral involvement may be under-recognised. The strong association with lower MoCA scores further highlights the need for routine cognitive assessment in SLE, particularly given the absence of a consensus definition for mild cognitive dysfunction despite its inclusion in the ACR NPSLE case definitions. Further studies to better understand the pathophysiological processes of NPSLE, including cerebrospinal fluid cytokine profiling and advanced neuroimaging modalities such as 18F-fluorodeoxyglucose uptake scans, may provide insights into the neuroinflammatory pathways contributing to cerebral injury in SLE and identify specific brain regions most susceptible to NPSLE.^1 32^ Collectively, the associations between cerebral atrophy, increasing age, lower education level, presence of NPSLE, longer disease duration and higher SDI scores support a model of cumulative antibody-mediated neuroinflammatory injury, resulting in axonal loss and cortical atrophy. These findings reinforce the importance of optimal disease control and management of vascular risk factors in the care of patients with SLE. As one of the few studies in a multi-ethnic Southeast Asian cohort using formal NPSLE attribution criteria, our results highlight the importance of early neurocognitive screening and proactive control of systemic inflammation to mitigate long-term cerebral injuries.

## Data Availability

Data are available on reasonable request.
